# Comparative assessment of phototherapy protocols for reduction of oxidative stress in partially transected spinal cord slices undergoing secondary degeneration

**DOI:** 10.1186/s12868-016-0259-6

**Published:** 2016-05-18

**Authors:** Bethany Eve Ashworth, Emma Stephens, Carole A. Bartlett, Stylianos Serghiou, Marcus K. Giacci, Anna Williams, Nathan S. Hart, Melinda Fitzgerald

**Affiliations:** Experimental and Regenerative Neurosciences, School of Animal Biology, The University of Western Australia, Crawley, WA Australia; Department of Biology and Biochemistry, The University of Bath, Bath, UK; Centre for Regenerative Medicine, University of Edinburgh, Edinburgh, UK; Department of Biological Sciences, Macquarie University, Sydney, NSW 2109 Australia

## Abstract

**Background:**

Red/near-infrared light therapy (R/NIR-LT) has been developed as a treatment for a range of conditions, including injury to the central nervous system (CNS). However, clinical trials have reported variable or sub-optimal outcomes, possibly because there are few optimized treatment protocols for the different target tissues. Moreover, the low absolute, and wavelength dependent, transmission of light by tissues overlying the target site make accurate dosing problematic.

**Results:**

In order to optimize light therapy treatment parameters, we adapted a mouse spinal cord organotypic culture model to the rat, and characterized myelination and oxidative stress following a partial transection injury. The ex vivo model allows a more accurate assessment of the relative effect of different illumination wavelengths (adjusted for equal quantal intensity) on the target tissue. Using this model, we assessed oxidative stress following treatment with four different wavelengths of light: 450 nm (blue); 510 nm (green); 660 nm (red) or 860 nm (infrared) at three different intensities: 1.93 × 10^16^ (low); 3.85 × 10^16^ (intermediate) and 7.70 × 10^16^ (high) photons/cm^2^/s. We demonstrate that the most effective of the tested wavelengths to reduce immunoreactivity of the oxidative stress indicator 3-nitrotyrosine (3NT) was 660 nm. 860 nm also provided beneficial effects at all tested intensities, significantly reducing oxidative stress levels relative to control (p ≤ 0.05).

**Conclusions:**

Our results indicate that R/NIR-LT is an effective antioxidant therapy, and indicate that effective wavelengths and ranges of intensities of treatment can be adapted for a variety of CNS injuries and conditions, depending upon the transmission properties of the tissue to be treated.

## Background

Neurotrauma encompasses spinal cord injury (SCI) and traumatic brain injury (TBI) and can involve damage to both grey and white matter of the CNS [1]. Secondary degeneration is a hallmark of neurotrauma, with spreading damage contributing to deteriorating structure and function [2]. Oxidative stress contributes to secondary degeneration [3] and occurs when excess reactive oxygen species (ROS) and/or reactive nitrogen species (RNS) overwhelm the endogenous antioxidant ability of the biological system [4]. While reactive species are essential for a range of normal cellular processes, excess leads to oxidative and nitrosative disruptions including protein nitration, DNA oxidation and lipid peroxidation [5–7], resulting in compromised oxidative metabolism, ATP depletion, necrosis, and dysregulated apoptosis [8]. If the excess production of reactive species can be limited, oxidative damage to DNA, lipids and proteins may be reduced and functional outcomes improved [9].

Red/near-infrared light therapy (R/NIR-LT), also known as photobiomodulation or phototherapy, is the therapeutic use of electromagnetic radiation at wavelengths characterized by relatively low energy densities in the red/near-infrared spectrum (600–1000 nm) [10]. R/NR-LT has been shown to have therapeutic effects in a range of CNS-specific injuries, resulting in improved functional recovery in cases of CNS injury [11], retinal degeneration [12, 13], stroke [14] and SCI [15] in rat models.

A proposed mechanism underlying R/NIR-LT therapy at the cellular level involves the activation of cytochrome c oxidase (COX) [14]. COX is complex IV of the mitochondrial respiratory chain and is considered to be one of the primary photoacceptors of visible and NIR light [16–18]. When COX absorbs photons at specific wavelengths (600–1000 nm), it undergoes a conformational change, altering its redox state, increasing its activity resulting in increased levels of ATP [19], and improved mitochondrial function [14]. Irradiation with 670 nm light has been demonstrated to increase COX activity and reduce oxidative stress in an in vivo model of secondary degeneration [20]; these effects consistent with improvements in oxidative metabolism. Additional proposed mechanisms of action for R/NIR-LT include reduced inflammation and release of nitric oxide from COX [14, 21, 22].

Evidence to date indicates that R/NIR-LT is an effective and safe antioxidant therapy in a range of preclinical models as well as in clinical settings [20]. However, the NEST-3 clinical trial for stroke, delivering 630/830 nm light using transcranial laser therapy, failed an interim futility analysis [23], perhaps owing to the failure to implement an effective, optimized treatment protocol. It is difficult to identify effective treatment parameters for clinical trials of R/NIR-LT given the large range of wavelengths, intensities, pulse structure treatment durations, treatment intervals and delivery methods that have been employed to date in pre-clinical studies and which alter actual dose delivered [22, 24, 25]. In particular, varying degrees of penetrance of light, dependent on wavelength can confound attempts to optimise treatment parameters and dissect out potential mechanisms of positive effects. Pre-clinical in vivo data to date indicate that 670 and 810–830 nm light are effective wavelengths [11, 26]. However, it is not yet clear whether efficacy is related to increased penetrance and thereby increased delivery of photons regardless of their delivery wavelength, or to specific effects of light at particular wavelengths. As such, light at short wavelengths may be even more effective than R/NIR wavelengths, if problems of penetration can be overcome.

Furthermore, effective parameters in one model do not necessarily apply to other models: a comparative study of R/NIR-LT efficacy in four CNS injury models in rat (partial optic nerve transection, light-induced retinal degeneration, TBI and SCI) indicated that it is necessary to optimize parameters individually for each type of CNS injury [26], perhaps due to differing penetrance of the light therapy. Therefore, to optimise treatment parameters of R/NIR-LT, a single, clinically relevant injury model where light can readily penetrate may assist.

Here, we adapt a well-established mouse organotypic spinal cord slice culture model which features the development of myelinated axons by 7 days in vitro (DIV) [27, 28] to rat, characterise the features of the culture system following partial transection injury and use it to determine the wavelengths and intensities of light therapy that can reduce an indicator of oxidative stress. A rat model was used in order to allow direct comparison to in vivo outcomes [26] in the same species. A model featuring three dimensional tissue architecture was desired as early attempts to optimise R/NIR-LT following excitotoxic insult to dissociated rat CNS cultures were unsuccessful [29]. Higher wavelengths of light are known to penetrate tissue more effectively due to the balance of increased absorption and reduced scattering [16, 30]. However, if it is merely delivery of photons that provides beneficial effects, shorter wavelengths may be effective in the cell culture context where penetrance through overlying tissue is not required. We therefore assess a range of wavelengths from 450 to 860 nm at low, medium and high intensities, where the medium intensity for each wavelength delivers an equivalent number of photons as that delivered by the popular VET75 LED array (Quantum Devices).

## Results

### Characterisation of injured spinal cord slice cultures from neonatal rat

**Fig. 1:**
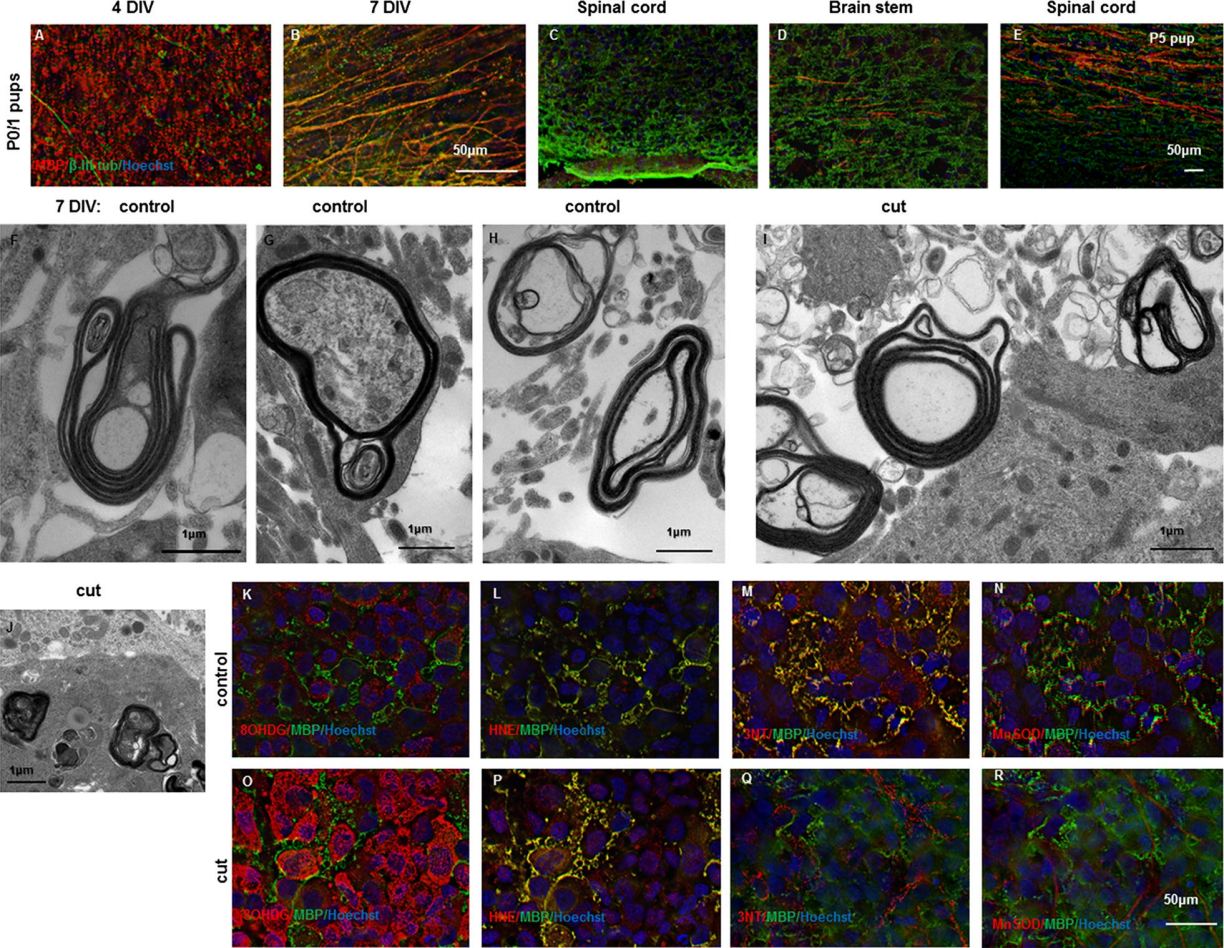
Characterisation of spinal cord slice cultures from neonatal PVG rat. Myelinated axons, indicated by β-III tubulin (*green*) and MBP (*red*) colocalisation, were not apparent in spinal cord slice cultures from P0–1 rat pups at 4 DIV (**A**) but were present at 7 DIV (**B**), scale = 50 µm for **A**, **B**. Immunohistochemical assessment of perfusion fixed spinal cord (**C**) and brain stem (**D**) from P0–1 rat pups showed no evidence of myelinated axons; myelinated axons were observed in spinal cord from P5 pups (**E**), scale = 50 µm for **C**–**E**. Electron microscopy of 7 DIV slice cultures revealed the presence of both relatively compact (**F**, **G**) and some decompacted myelin sheaths (**H**) in uncut slice cultures; cut injury 24 h previously resulted in increasingly decompacted myelin (**I**) and aggregates of myelin debris (**J**), scale = 1 µm. Immunoreactivity of 8OHdG (**K**) increased following cut injury (**O**), as did immunoreactivity of HNE (**L**, **P**), 3-NT (**M**, **Q**) and MnSOD (**N**, **R**). Immunoreactivity of all oxidative stress indicators is shown in* red*, together with MBP immunoreactivity (*green*) and Hoechst nuclear stain (*blue*); representative images adjacent to the cut injury are shown. Note that multiple* colour* immunofluorescence was employed and as such, 8OHDG and HNE images show the same field of view, as do 3NT and MnSOD images, scale = 50 µm

Spinal cord slices were prepared from postnatal day (P) 0–1 day old Piebald Virol Glaxo (PVG) rat pups. In P0–1 pups the uninjured spinal cord slice cultures contained cells that were myelin basic protein (MBP) immunopositive but had no myelinated axons at 4 DIV (Fig. 1A); some axons were myelinated by 7 DIV (Fig. 1B). Immunostained spinal cord sections collected from PVG rat pups similarly showed no myelinated axons in the uninjured spinal cord (Fig. 1C) or brain stem (Fig. 1D) at P0–1; or of P2 pups (not shown). Myelinated axons were, however, apparent in spinal cord sections at P5 (Fig. 1E), indicating that the development of myelination in neonatal rat in vitro parallels events in vivo. It is important to note that the myelination of axons in the uninjured rat spinal cord slice cultures was variable and that myelination was not always apparent. Electron microscopy revealed that myelin was spirally wrapped around some axons, but compaction of myelin was not always complete (Fig. 1F–H). Myelination was most consistent in cultures derived from P0–1 rat pups and this age of pup was chosen for further analyses.

Spinal cord slice cultures from P0–1 rat pups were subjected to a cut injury after 7 DIV. Approximately half of the width of the slice was transected with a scalpel, resulting in transection of axons within the slice, as ascertained by subsequent immunohistochemical analyses. At 1 day following injury, myelinated axons were observed less frequently than in uninjured control slices; myelin was often decompacted (Fig. 1I) and aggregates of myelin debris were present (Fig. 1J). Cut injury resulted in increased immunoreactivity of a range of indicators of oxidative stress in the slice cultures 24 h following insult, both adjacent to the cut area and more distally (Fig. 1K–R). 8-hydroxyguanosine (8OHDG) immunoreactivity, indicative of oxidative damage to DNA was increased in cell bodies, and as expected, was not co-localised with MBP (Fig. 1K, O); the area immediately adjacent to the cut injury is shown (Fig. 1O). Note that immunoreactivity of MBP was not clearly aligned along axons and in places was reminiscent of myelin debris (Fig. 1K–R). As noted above, myelin debris was also observed by electron microscopy in cut injured cultures (Fig. 1I, J). 4-Hydroxynonenal (HNE) immunoreactivity, indicative of lipid peroxidation, was increased and co-localised with MBP immunoreactivity (Fig. 1L, P), as expected given the high lipid content of myelin. Increased immunoreactivity of 3-nitrotyrosine (3NT), indicative of protein nitration by oxidative species, serves as a marker for oxidative/nitrosative stress in a range of cell types including myelinating oligodendrocytes [3]. Immunoreactivity of 3-NT was widespread in the tissue section and immunointensity was slightly increased following cut injury; staining was not apparent in MBP positive areas after injury (Fig. 1M, Q). Similarly, immunoreactivity of manganese superoxide dismutase (MnSOD), an antioxidant enzyme which converts superoxide to hydrogen peroxide, was slightly increased following injury and seldom co-localised with MBP immunoreactivity, particularly following cut injury (Fig. 1N, R).

**Fig. 2:**
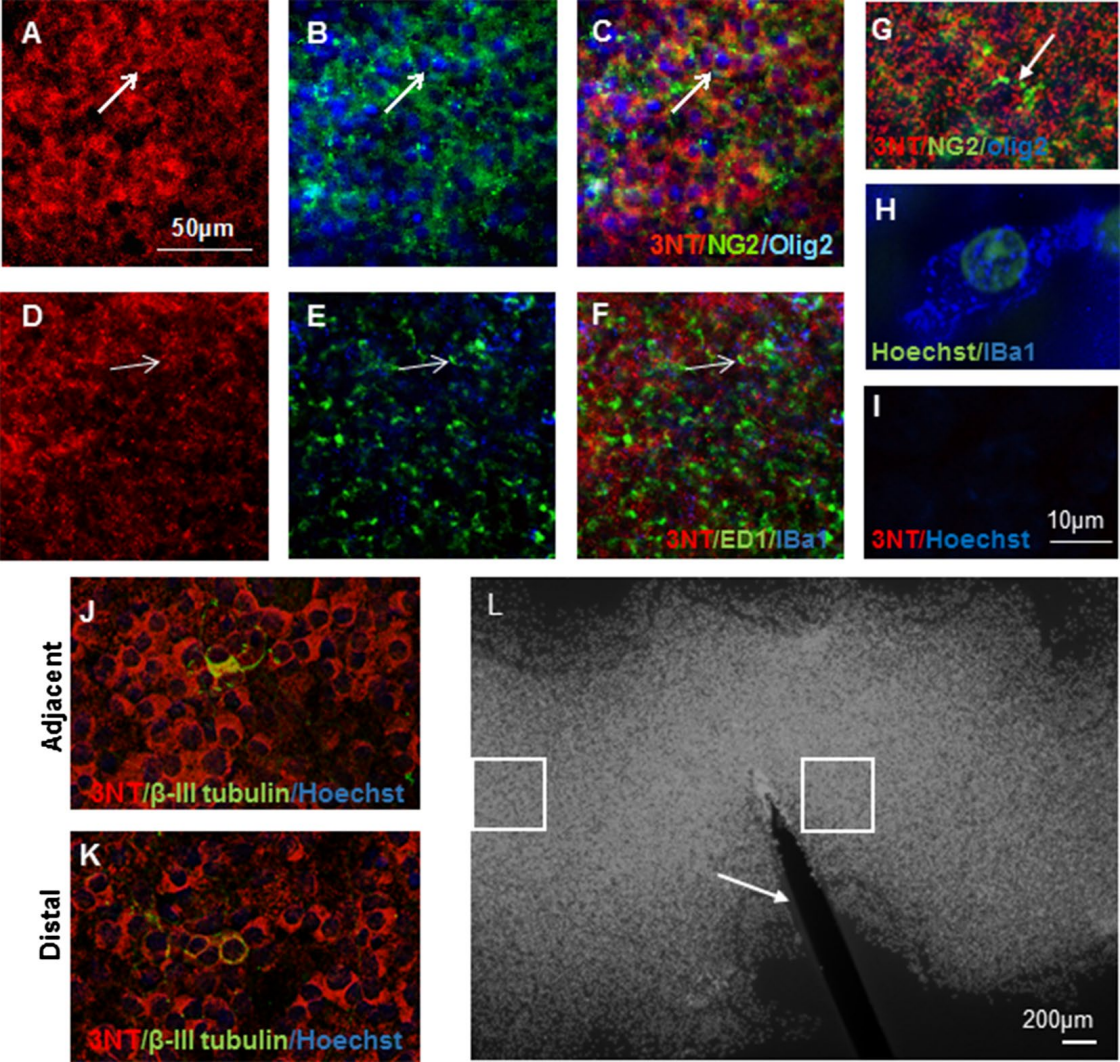
Representative images of 3NT immunoreactivity (**A**, **D**), colocalised with some NG2/Olig2 + ve cells (**B**, *arrow*) with overlay (**C**), and some ED1/IBA1− +ve cells (**E**, *arrow*) with overlay (**F**). Higher magnification images of NG2/Olig2+ (**G**) and IBA+ (**H**) cells are provided, together with a control image omitting the 3NT primary antibody (**I**). 3NT immunoreactivity was approximately equivalent both adjacent to the cut injury (**J**) and more distally (**K**); 3NT (*red*), β-III tubulin (*green*), Hoechst nuclear stain (*blue*). Scale = 50 µm for images **A**–**F**, **J**, **K**; scale = 10 µm for **G**–**I**. A representative image of a spinal cord slice with cut injury, stained with Hoechst nuclear stain (**L**); *boxed areas* indicate analysis sites close and distal from the injury (indicated by an *arrow*), scale = 200 µm

Additional immunohistochemical analyses were conducted to ascertain whether 3NT colocalised with other cell types within the organotypic spinal cord slice cultures. 3NT immunoreactivity was broadly expressed throughout the spinal cord slice cultures (Fig. 2A, D), with some colocalisation with NG2^+^/Olig2^+^ oligodendrocyte precursor cells (Fig. 2A–C, g arrows). 3NT occasionally colocalised with IBA1^+^ microglia and ED1^+^ microglia/macrophages (Fig. 2D–F, h arrows); a control image where the primary antibody was omitted is provided (I). 3NT immunoreactivity was not different when comparing areas immediately adjacent to the cut injury, to areas more distal (Fig. 2J, K). The data indicate that the spread of oxidative/nitrosative stress occurred relatively rapidly, such that damage was uniformly distributed by 24 h after the injury. Given that the spinal cord slice cultures are approximately 4 mm in length, 3NT immunoreactivity spread more than 1.5 mm in 24 h of culture (L). While the size of spinal cord lesions in vivo is highly dependent upon the nature and severity of the injury, our data indicate the potential for significant spread of oxidative/nitrosative damage.

**Fig. 3:**
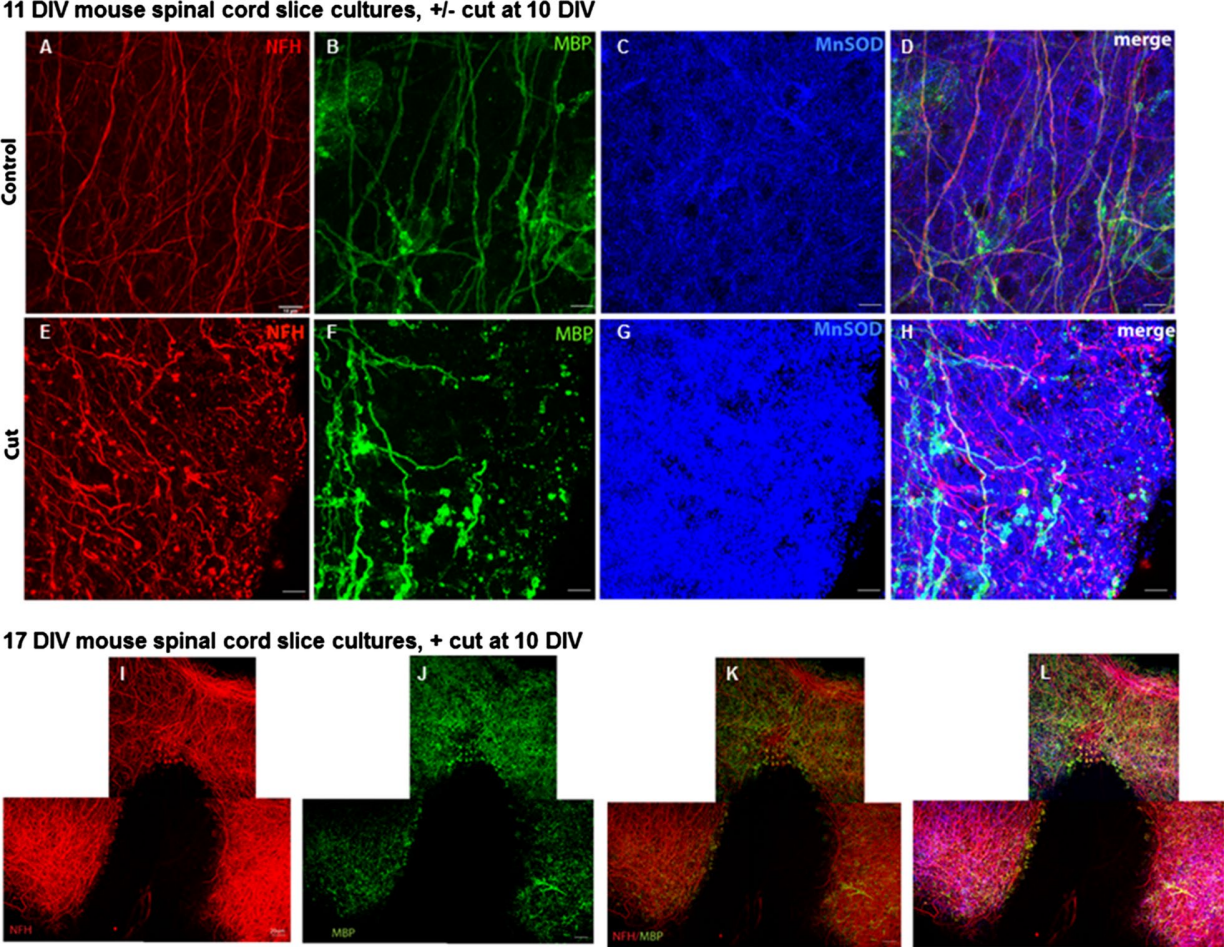
The effects of cut injury on spinal cord slice cultures from neonatal mouse. Spinal cord slice cultures were cultured for 10 DIV, and either not cut (**A**–**D**), or subjected to cut injury (**E**–**H**). Immunohistochemical analysis of NFH (*red*), MBP (*green*) and MnSOD (*blue*) with overlay in uncut and cut cultures are shown at 11 DIV (**A**–**H**), scale = 10 µm. Immunohistochemical analysis of NFH (*red*), MBP (*green*) with overlay (**I**–**K**) and overlay with Hoechst (**L**) in cut cultures are shown at 17 DIV (**I**–**L**), scale = 20 µm

As a comparison, spinal cord slice cultures were prepared from P0–1 mouse pups, cultured for 10 DIV and then subjected to cut injury. Myelinated axons were clearly apparent in uninjured cultures (Fig. 3A, B). While lengths of MBP positive myelin were present 1 day after cut injury, myelin debris was apparent (Fig. 3E, F). By 7 days after the cut injury, some MBP immunoreactivity colocalised with neurofilament positive axons in the uncut section, but cut axons present in the lower half of the image were unmyelinated (Fig. 3I–L). MnSOD immunoreactivity was increased, particularly adjacent to the cut site (Fig. 3C, G), indicating oxidative stress that may be contributing to demyelination in the area with transected axons and reduced myelination. Despite some demyelination in the cut injured mouse slice cultures, it is apparent that myelination was more robust in the mouse slice cultures than in those from rat.

### Light treatment reduced an indicator of oxidative stress

**Fig. 4:**
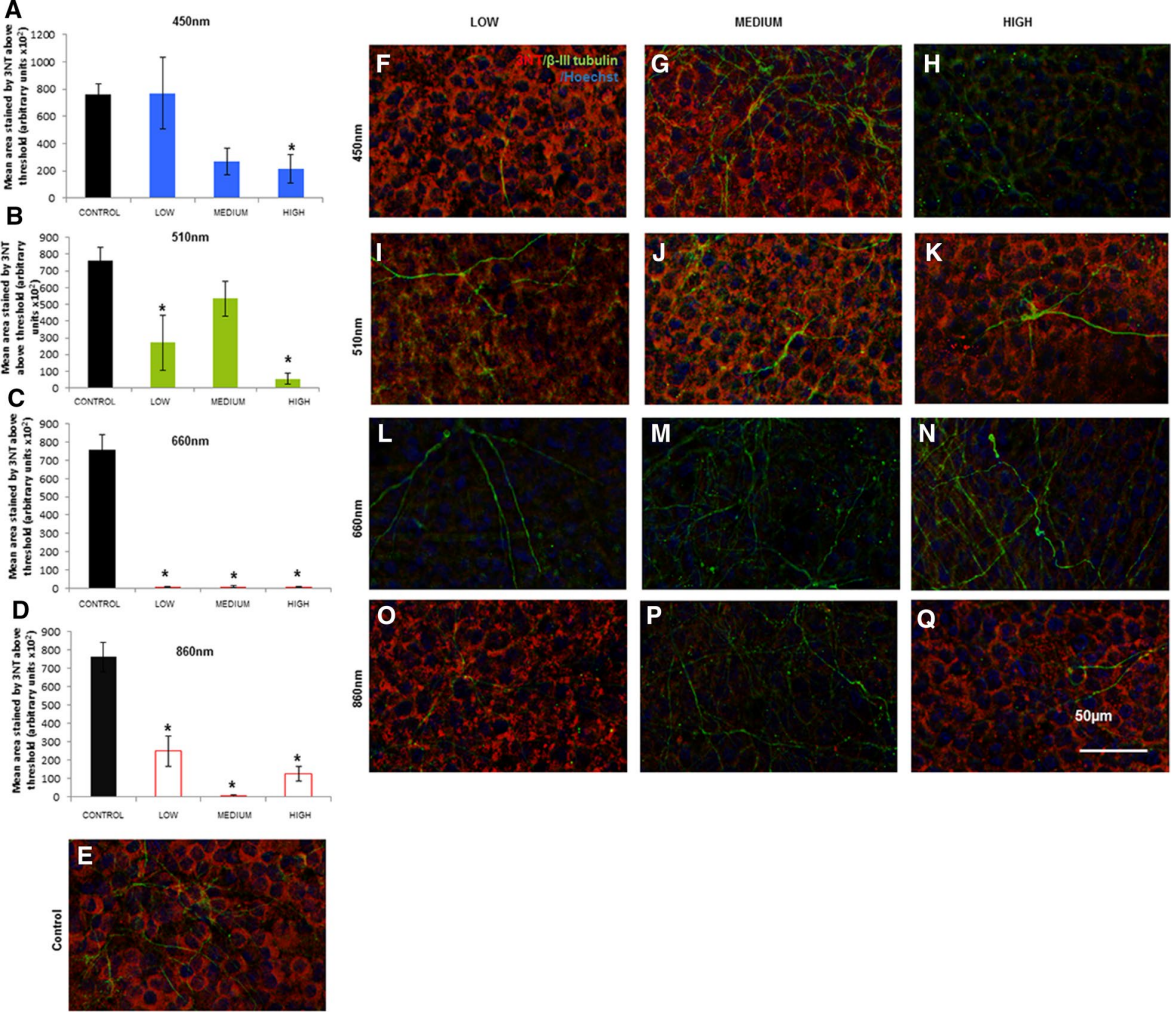
3-NT immunoreactivity of spinal cord slice cultures following treatment with light of varying wavelengths and intensities. **A**–**D** Quantification of the area of 3-NT immunoreactivity above a set threshold in spinal cord slice cultures treated with 450, 510, 660 or 860 nm light. Low (1.93 × 10^16^), intermediate (3.85 × 10^16^), or high (7.70 × 10^16^) (photons/cm^2^/s) intensities were delivered. Significant differences from control for each intensity of light delivered are indicated by *(p ≤ 0.05). **E**–**Q** Representative images are provided for each treatment intensity and wavelength, imaged immediately adjacent to the cut: 3-nitrotyrosine immunoreactivity (3NT) is red, together with β-III tubulin immunoreactivity to identify axons (*green*) and Hoechst nuclear stain (*blue*) for control (**E**), 450 nm treated (**F**–**H**), 510 nm treated (**I**–**K**), 660 nm treated (**l**–**N**) or 860 nm treated (**O**–**Q**) cultures. Scale = 50 µm for all images. Each experiment was conducted three times and data from a single experiment with 8 replicates per group are shown

**Fig. 5:**
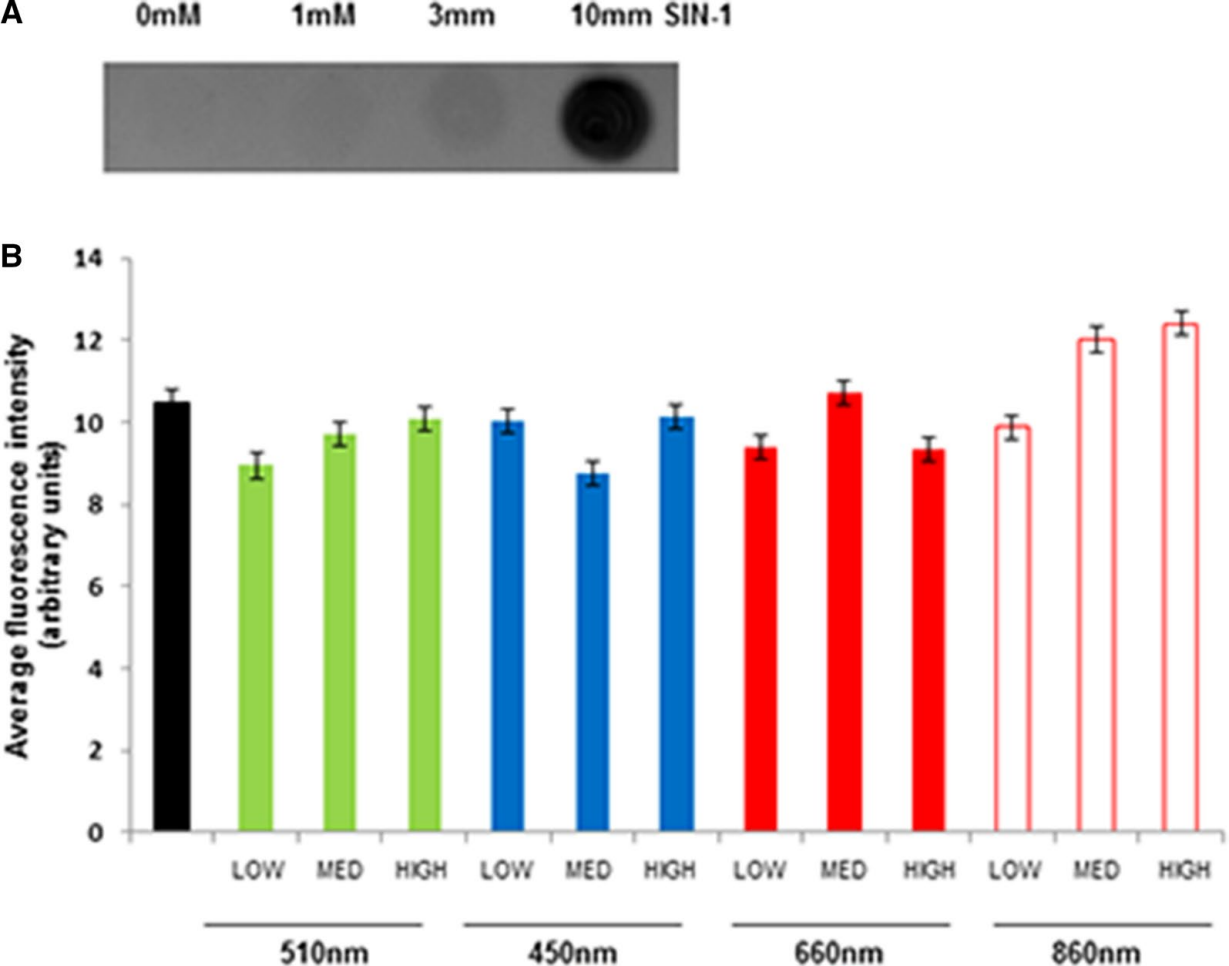
3NT immunoreactivity in SIN-1 treated PC12 cells and 8OHDG immunoreactivity of spinal cord slice cultures following treatment with light of varying wavelengths and intensities. **A** PC12 cell supernatant 3NT immunoreactivity following treatment with 1, 3 or 10 mM SIN-1 relative to untreated control cells. **B** Quantification of the area of 8OHDG immunoreactivity above a set threshold in spinal cord slice cultures treated with 450, 510, 660 or 860 nm light. Low (1.93 × 10^16^), intermediate (3.85 × 10^16^), or high (7.70 × 10^16^) (photons/cm^2^/s) intensities were delivered. There were no significant differences between control and treatment groups. The experiment was conducted three times and data from a single experiment with 8 replicates per group are shown

We used the partially transected rat in vitro spinal cord slice culture model system to compare effects of treatment with a range of wavelengths and intensities of light on oxidative stress, in tissue vulnerable to secondary degeneration. The slice cultures were injured at 7 DIV and tissue was assessed 24 h after cut injury (8DIV), immediately following two, 30 min light or control treatments 24 h apart. Indicators of oxidative stress examined were 3NT, HNE and 8OHDG. In 450 nm treated spinal cord slices we detected a significant decrease in the area of 3NT immunoreactivity above a set threshold at the intermediate and high intensities relative to control (p < 0.05) (Fig. 4A, E–H), suggesting dose dependence at this wavelength. Immunoreactivity of 510 nm treated slices was significantly reduced at both low and high intensities of light compared to control (p < 0.05) (Fig. 4B, E, I–K). In contrast, 660 nm light significantly reduced 3NT immunoreactivity consistently at all three tested intensities (p ≤ 0.05) (Fig. 4C, E, L–N). Similarly, 860 nm light significantly reduced 3-NT immunoreactivity at all three intensities compared to control (p ≤ 0.05) (Fig. 4D, E, O–Q), albeit to a lesser extent than 660 nm light and with a trend to a biphasic effect. Note that there were no statistically significant differences between the varying treatment intensities for 860 nm, or 660 nm light (p > 0.05). Specificity of the 3-NT antibody was demonstrated using established procedures [31], with increased 3-NT immunoreactivity in the cell culture supernatant of rat pheochromocytoma (PC12) neural like cells following treatment with SIN-1 (morpholinosydnonimine), a peroxynitrate generator [32], relative to untreated control (Fig. 5a). The effects of the full range of wavelengths and intensities on immunoreactivity of 8OHDG and HNE were assessed following cut injury in the slice cultures. There were no significant effects of any of the wavelengths and intensities of light on 8OHDG immunoreactivity, when considering both the area above a set threshold of intensity or the mean fluorescence intensity of the image (Fig. 5b, mean fluorescence intensity shown). Similar results were observed for HNE (not shown). Taken together our data indicate differential effects of R/NIR-LT on oxidative damage, with beneficial effects on oxidative/nitrosative damage to protein, but not damage to DNA or lipid. A summary table of immunohistochemical outcomes of the study is provided in Table 1.

**Table 1:** Summary of immunohistochemical outcomes

	Increased with cut	Co-localisation with MBP	Co-localisation with microglia/macrophages, OPCs	Effect of light treatment
8OHDG	++	**−**	ND	**−**
HNE	+	+	ND	**−**
3NT	+	**−**	+	↓
MnSOD	+	**−**	ND	ND

+ Is increased immunoreactivity, ++ is strongly increased immunoreactivity, − is negative or no effect, ND is not assessed, ↓ is decreased immunoreactivity

## Discussion

R/NIR-LT is a prospective antioxidant therapy for preserving function following CNS injury. The treatment is comparably cheap, non-invasive and easy to administer when using LED arrays, and has shown promise in numerous pre-clinical models of CNS injury and for some indications in clinical trials for stroke [20, 24]. However, lack of optimization using comparative studies has probably contributed to the failure of widespread clinical use of R/NIR-LT [24], and there is a need for a standardized, consensus treatment protocol. In this study, we have firstly developed and characterised an in vitro rat model of CNS injury suitable for optimizing treatment strategies for neurotrauma. Secondly we have used the model to compare a range of wavelengths and intensities for efficacy at reducing oxidative stress, in an effort to provide data useful for optimisation of light therapy, with the added advantage of a reduction in animal numbers required. The intensities of light delivered were designed to encompass those shown to be effective in vivo once penetrance of light has been accounted for [39] and were standardised for the number of photons that reached the surface of the cultures for each wavelength utilised. As such, outcomes between wavelengths are directly comparable. Our data suggest 660 nm light to be the most promising wavelength of those tested for reducing oxidative stress, performing consistently and effectively across all three intensity settings.

When light interacts with biological tissue, photons are either absorbed by cellular constituents or scattered [33], with optimal penetration occurring within an “optical window” [34]. Penetration is dependent on absorption and scattering [33], both of which are greater at shorter, blue/green wavelengths [35]. However, in the current in vitro study, penetrance of the different wavelengths is likely to be relatively uniform and the effects of equivalent numbers of photons of different wavelengths were directly assessed. Different wavelengths of light have been shown to have different effects upon photoacceptors. For example, wavelength bands of 330, 404–420, 680 and 825 nm [36, 37] have been shown to result in oxidation of COX, and distinct wavelength bands of 450, 620 and 760 nm reduce COX [36]. Our observation of increased COX activity in vivo following 670 nm treatment [38], indicate that oxidation of COX by red light increases the activity of the enzyme, at least associated with, and perhaps mediating the therapeutic effect. The results of the current study demonstrating that 660 nm was the most effective of the tested wavelengths at reducing oxidative stress support these findings and indicate that oxidation of COX may mediate beneficial therapeutic effects.

The other tested wavelengths were outside the optimal wavelengths for COX oxidation, and in the case of 450 nm were likely to lead to reduction of the enzyme, perhaps accounting for the limited anti-oxidant efficacy. Nevertheless, the highest intensity of light tested in the current study still reduced 3NT immunoreactivity at all four tested wavelengths. Absorption of sufficient energy by COX to enable conformational change may have resulted in release of nitric oxide from COX, [14] thereby leading to beneficial effects of wavelengths of light that reduce COX. An alternative mechanism may involve the flavoprotein NADH dehydrogenase (NADH-D), which has been suggested as a photoacceptor involved in blue light therapy [39]. Its action spectra peak at 448 nm [40, 17] corresponds to the blue wavelength setting used in this study (450 nm). Although activation of NADH-D [39, 41] may promote electron drive through the electron transport chain, this therapeutic effect may be overwhelmed by the detrimental accumulation of superoxide production, perhaps explaining why blue light has produced results reflective of lower efficacy in the current study. It is interesting to note that of the oxidative stress measures assessed, light therapy only resulted in decreases in 3NT immunoreactivity, indicating that oxidative/nitrosative damage to proteins may be limited by the mechanisms described above, whereas damage to DNA and lipid oxidation are not. Assessment of anti-inflammatory effects of the various wavelengths in the current model would also be interesting to address.

Measures of oxidative damage in the current study rely upon the specificity of the commercial antibodies employed. It is challenging to confirm specific immunostaining of oxidative or nitrosative damage: for example, increased nitrotyrosine immunoreactivity in tissue reflects nitration of tyrosines in a large range of proteins in response to oxidative and nitrosative stress. As such, Western analysis of binding of the antibody will result in a large number of bands and commercial descriptions of these antibodies do not show Western blots. Nevertheless, our observation of increased 3-nitrotyrosine immunoreactivity in the supernatant of cells treated with the peroxynitrate generator SIN-1 [31] indicates that changes in immunoreactivity occur in response to reactive nitrogen species.

Complete transection of the spinal cord, although reflective of complete SCI in patients [42], is rarely seen in the clinic [43, 44] and partial transection models are more reflective of clinical SCI. Furthermore, partial transection models are valuable for assessing axonal regeneration as an additional measure in SCI [45, 46], and are increasingly used to assess the effects of combinatorial therapies on axonal regeneration post-injury [47]. Our data suggest 660 nm as an effective wavelength setting for light therapy. However in order to ensure delivery of sufficient intensity of light at this wavelength to injured tissue in vivo, it is necessary to assess transmission of the light through the overlying tissue. Calculated and measured assessments of penetrance of irradiation to the spinal cord range from 6 to 11.3 % depending upon wavelength [11, 24, 26]. Together with data from the current study, these values can be used to calculate likely effective dosages of R/NIR-LT to SCI. The lowest effective intensity of 660 nm light tested here was 1.93 × 10^16^ photons/cm^2^/s. We have measured that the VET75 LED array delivers 5.3 × 10^16^ photons/cm^2^/s to the surface of the skin when held at 3 cm above the skin [26]. Given 6.6 % penetrance, we calculate that only approximately 0.35 × 10^16^ photons/cm^2^/s penetrate to the SCI, perhaps explaining our observed lack of effect of R/NIR-LT following SCI in vivo [26]. The nature of individual CNS injuries may impact upon efficacy of R/NIR-LT, with differing disease pathologies likely differentially modulated by altered photoacceptor activity. For example, increased glial scarring and immune cell infiltration in SCI may overwhelm positive effects of R/NIR-LT on oxidative stress. Nevertheless, it is apparent that transmission of R/NIR-LT must be carefully considered when designing effective R/NIR-LT treatment protocols for CNS injuries.

## Conclusions

Our comparative assessment has resulted in the identification of effective protocols of R/NIR-LT that can be adapted to individual CNS injuries, bearing in mind the transmission properties of the injured and overlying tissue. We have demonstrated that 660 nm red light is the most effective of the tested wavelengths at reducing oxidative stress, likely due to oxidation of COX. R/NIR-LT at 860 nm also significantly reduced oxidative stress at all tested intensities. We conclude that although R/NIR-LT appears to be a relatively simple therapy, the choice of dose regime and wavelength utilised needs as much care and attention as designing drug dosing protocols.

## Methods

All procedures involving animals were approved by the University of Western Australia Animal Ethics Committee, Approval number RA3/100/673, or the UK Home Office project licence number 60/4524 and adhered to the National Health and Medical Research Australian Code of Practice for the care and use of animals for scientific purposes. Neonatal PVG rat pups (P0–P5), sex not specified, were procured from the Animal Resources Centre (Murdoch, Western Australia). The mother and pups were housed under standard conditions including 12 h light/dark cycles and ad libitum access to chow and water.

### Spinal cord collection and culture

Rat or mouse pups at P0–1 days old (n = 6/experiment) were euthanized using pentobarbital (Virbac, Australia Pty. Ltd., NSW, Australia), administered intraperitoneally at a dose of 800–1000 mg/kg (~0.02 to 0.03 ml) as per standard best practice. Cultures were generated from six pups per experiment to ensure sufficient spinal cord slice cultures were available for all experimental groups; cultures were randomly allocated to experimental groups. Each experiment was repeated at least three times. Spinal cords were removed and a 5 mm segment of cervical-thoracic spinal cord dissected into spinal growth culture media (containing 50 % minimal essential medium, 25 % heat-inactivated horse serum, 25 % Earl’s balanced salt solution, supplemented with 1 % pen/strep, 1 % glutamax, 0.5 % fungizone, 1.4 % d-glucose). Spinal cords were placed with the posterior surface uppermost and 200 μm (for rat) or 300 µm (for mouse) longitudinal slices generated using a Mcllwain Tissue Chopper. Three slices were transferred to each Millicell-CM low height cell culture membrane (Millipore, Germany) and cultured in six-well plates; each well containing 1 ml spinal growth culture media. The spinal slices were cultured for 7–17 days at 37 °C 5 % CO_2_, with fresh spinal media supplied every second day. The media change after 6 DIV incorporated 24.8 mM Hepes into the spinal cord media to buffer pH changes during light therapy. Note that myelinated axons were not observed in slice cultures prepared from P3–4 day old rat pups at 4, 7, 10, 17 or 24DIV (not shown) and cultures from pups of this age were not examined further.

### Transection of cultures and R/NIR light treatment

Phototherapy was administered to the organotypic spinal slices using custom-built LED arrays. The four individual narrow-band LED arrays delivered light with centre wavelengths of 449, 516, 662 and 865 nm (referred to hereafter as 450, 510, 660 and 860 nm, respectively). The emitted light intensity of each device was controlled by adjusting the current supplied to the LEDs and measured using a calibrated CCD spectroradiometer (USB 4000, Ocean Optics) fitted with a cosine collector, allowing calculation of the total number of photons delivered at all emitted wavelengths using the Spectrasuite software program (Ocean Optics). The light output of each LED device was adjusted to produce equal photon irradiance when delivered at a fixed distance of 5 cm above the spinal slices, with the LED array fixed above the cultures to allow uniform illumination while not touching the culture dish. Light doses were delivered to cultures at three intensities [1.93 × 10^16^ (low), 3.85 × 10^16^ (intermediate) and 7.70 × 10^16^ (high) photons/cm^2^/s] at each nominal wavelength. Sham-treated control spinal cord slices were subject to identical treatment conditions, but the overlying LED device was not switched on. Lower intensities were delivered prior to higher intensities to ensure residual heat was not a confounding factor in low intensity treatments. All cultures were also exposed to normal laboratory lighting conditions during the treatment process; the culture incubator is a dark environment.

After 7 DIV a #11 scalpel blade was used to make a full depth cut partway through the width of the slice, at the midpoint of each slice, to induce a partial transection insult. Light treatment was applied to the membranes containing the slice cultures for a duration of 30 min, the first treatment delivered immediately following insult and the second 24 h following insult. Temperature was monitored and maintained at 37 °C throughout treatment using an incubator, whilst pH was controlled by the prior addition of Hepes (24.76 mM) to the media. Immediately following the second 30-min light treatment, slices were fixed on the culture membranes for 30 min with 4 % paraformaldehyde in 0.1 M phosphate buffer and washed with phosphate buffered saline (PBS). Slices on membrane were cut out and stored at 4 °C in a 24 well plate containing PBS azide (0.1 % sodium azide in sterile PBS, 1 ml/well) for future immunohistochemical analyses.

### Immunohistochemical analyses

Immunohistochemical analyses were conducted on spinal slices still adhered to the membranes according to established procedures [48], using primary antibodies recognizing: 3NT(1:500; Abcam, Cambridge, UK); β-III Tubulin (1:500; Covance); neurofilament (NFH) (1:50,000; Encor); 8OHDG (1:500; Abcam, Cambridge, UK); HNE (1:200; Jomar Bioscience); MnSOD (1:500, StressGen); MBP (1:500, Abcam); Olig2 (1:500; Abcam); NG2 (1:250; Invitrogen); IBA1 (1:500; Novachem), ED-1 (1:500; Millipore) incubated overnight with the slices at 4 °C. Secondary antibodies were species-specific AlexaFluor^®^ 555- and 488-conjugated (1:400; Invitrogen), with the addition of Hoechst nuclear stain (1:1000; Invitrogen, VIC); incubated for 2 h at room temperature.

The specificity of the 3NT antibody was assessed using PC12 cells grown in RPMI medium supplemented with 10 % horse serum, 5 % fetal bovine serum, 2 mM l-glutamine, 2 mM penicillin–streptomycin, 1 mM MEM sodium pyruvate and 0.1 mM MEM non-essential amino acids (GIBCO ^®^, Invitrogen, Carlsbad, California, USA), on flasks coated with poly-l-lysine (PLL; 10 μg/ml; Sigma, St Louis, Missouri, USA) and housed in an incubator at 37 °C, 5 % CO_2_. Cells were seeded at 4 × 10^4^ cells/well of a PLL coated 24 well plate, allowed to adhere and then incubated with 1, 3 or 10 mM SIN-1 (Sigma-Aldrich) for 24 h. 5 µl samples of resultant cell culture supernatants were assessed by dot-blot.

### Microscopy and image analysis

For each experiment, intensity of immunoreactivity was visualized and imaged in a single session using a Nikon Eclipse Ti Inverted Microscope (Nikon Corporation, Japan) or a Leica SP7 confocal: images were captured at constant exposure settings across all treatment groups. Each experiment was repeated at least three times, using cultures prepared on different days. Images were captured at areas both close and distal to the injury site, with two close and two distal images taken per slice. Two spinal cord slices were imaged per treatment group, generating eight data points for each group. Each image consisted of a stack of 13 visual slices captured at 0.5 μm increments along the z-axis, and obtained from the middle 6 μm of the slice. Images captured using the non-confocal microscope were deconvoluted using autoquant blind deconvolution with Nikon Elements AR software. Image analysis was conducted on a single central visual slice using ImageJ/Fiji analysis software, setting constant arbitrary threshold intensities for all images in an analysis and determining the area above the threshold intensity and the mean intensity of fluorescence of the image.

### Statistical analysis

All data are displayed as mean ± standard error of the mean (SEM). Statistical analyses were conducted using SPSS statistical software. Data from regions both close and distal to the transection were combined, as statistical analysis using nonparametric Wilcoxon paired-samples test revealed no significant differences between the regions (p ≤ 0.05). Equality of variance F-tests were conducted to test for homogeneity of variance in groups within experiments. Collated data were analysed by comparing treated to control using analysis of variance (ANOVA) with Games Howell post hoc test at 95 % confidence intervals (p ≤ 0.05). Statistical assessments were conducted independently for each experiment and representative analyses shown; it is not possible to average immunohistochemical intensity data from different experiments due to inter-experiment variability in baseline values.

## Abbreviations

ANOVA: analysis of variance
CNS: central nervous system
COX: cytochrome *c* oxidase
DIV: days in vitro
HNE: hydroxynonenal
8OHDG: 8-hydroxyguanosine
LED: light emitting diode
MBP: myelin basic protein
MnSOD: manganese superoxide dismutase
NADH-D: NADH dehydrogenase
NEST: NeuroThera Effectiveness and Safety Trial
NFH: neurofilament
3NT: 3-nitrotyrosine
P: postnatal day
PBS: phosphate buffered saline
PVG: Piebald Virol Glaxo
RNS: reactive nitrogen species
ROS: reactive oxygen species
R/NIR-LT: red/near-infrared light therapy
SCI: spinal cord injury
SEM: standard error of the mean
TBI: traumatic brain injury
